# Environmental health practitioners potentially play a key role in helping communities adapt to climate change

**DOI:** 10.1186/s12889-018-6378-5

**Published:** 2019-01-11

**Authors:** Busisiwe Shezi, Angela Mathee, Wellington Siziba, Renée A. Street, Nisha Naicker, Zamantimande Kunene, Caradee Y. Wright

**Affiliations:** 1 Environment and Health Research Unit, South African Medical Research Council, Durban, South Africa; 2 Environment and Health Research Unit, South African Medical Research Council, Johannesburg, South Africa; 3 Faculty of Health Sciences, University of Johannesburg, Johannesburg, South Africa; 4 University of the Witwatersrand, Johannesburg, South Africa; 5 Department of Occupational and Environmental Health, University of KwaZulu-Natal, Durban, South Africa; 6 The Epidemiology and Surveillance Section, National Institute for Occupational Health, National Health Laboratory Services, Johannesburg, South Africa; 7 Environment and Health Research Unit, South African Medical Research Council, Pretoria, South Africa; 8 Department of Geography, Geo-informatics and Meteorology, University of Pretoria, Pretoria, South Africa

**Keywords:** Climate change, Environmental health, South Africa, Health professionals

## Abstract

**Background:**

Vulnerable population groups in South Africa, especially those living in poverty, young children, women, the elderly and people with pre-existing diseases, are susceptible to new or exacerbated health threats resulting from climate change. Environmental Health Practitioners (EHPs) can play an important role in helping communities adapt to climate change health impacts, however, effective coordination of this requires further understanding of their roles in implementing climate change-related adaptation actions in communities.

**Methods:**

A cross-sectional survey using convenience sampling was undertaken at the January 2017 conference for EHPs hosted by the South African Institute of Environmental Health in Cape Town. All EHPs who attended the conference were invited to complete a study questionnaire that requested information on participant demographics, as well as climate change related-knowledge, practices and perceptions.

**Results:**

Majority of participating EHPs (*n* = 48; 72.8%) had received formal or informal training on climate change and health. Thirty-nine percent of EHPs indicated that they had a climate change and health-related committee / working group in their department, a policy or strategy (41.0%) and budget allocated for climate change and health-related work (51.5%). A total of 33.3% had participated in climate change-related projects. Majority (62.2%) of EHPs believed that they should play a supportive role in addressing climate change while 37.8% believed that EHPs should play a leading role.

**Conclusions:**

Recognising the need for raising awareness about climate change adaptation as well as implementing appropriate interventions to combat climate-related ill health effects, especially among vulnerable groups, EHPs are well-placed to adopt significant roles in helping communities to adapt to climate change.

## Background

The Intergovernmental Panel on Climate Change (IPCC), the World Health Organization and other international institutions have indicated that climate change will exacerbate or redistribute current environmental health risks [1, 2]. Vulnerable population groups in South Africa, especially those living in poverty, children, women, the elderly and people with pre-existing diseases, are susceptible to health threats resulting from climate change, such as increasing temperature, floods and droughts [3–5]. Little is known about the coping capacities of vulnerable sub-populations with respect to these climate change-related environmental effects [6]. Environmental Health Practitioners (EHPs) play an important role in local implementation of adaptation strategies, particularly in communities with vulnerable populations. Equally so, climate change adaptation tends often correspond more closely to environmental health practice at a local rather than global level [7].

IPCC defines Southern Africa as a region likely to experience an increase in temperature that exceeds the global average [1]. In South Africa the average annual temperatures are reported to have increased by 1.5 °C times the observed 0.65 °C over the past five decades [8]. The frequency in extreme rainfalls has also increased [8]. It is predicted that interior regions of South Africa will warm by a further ~ 3–4 °C by 2100 [8].

South Africa has a population size of 57 million [9]. EHPs deal with the environmental safety and health of communities in the local sphere of government [10]. Each of the nine provinces are managed by local government agencies, namely district and metropolitan municipalities [11]. The largest urbanized areas are governed by metropolitan municipalities, while the rest of the country is divided into district municipalities, each of which consists of several local municipalities [11]. There are 228 local municipalities grouped into 44 district municipalities and eight metropolitan municipalities [11]. According to SA Health Review (2017), the ratio of EHPs per 100,000 target population by province ranges from 0.4 to 2.3 [10]. EHPs are registered with the Health Professions Council of South Africa (HPCSA) and in 2015, there were 3585 registered EHPs [10]. The work of EHPs is defined in their Scope of Practice of Environmental Health and includes water quality, food control, waste managements, health surveillance of premises, surveillance and prevention of communicable diseases, vector control and environmental pollution control [12]. Although the Scope of Practice does not explicitly mention climate change and health-related adaption action [13], their core work areas are increasingly affected by climate change.

In 2011, the Conference of the Parties (COP)-17 meeting hosted in South Africa stipulated the importance of involving EHPs, health care professionals and health officials in helping communities to increase their resilience and capacity to respond to, and withstand the impact of climate change [8]. In South Africa, the National Climate Change Response White Paper [14] outlines the National Department of Health’s (NDOH’s) responsibilities related to mitigation and adaptation to climate change and emphasises the role of EHPs as part of this effort. Furthermore, South Africa’s National Climate Change and Health Adaptation Plan [13] calls for EHPs to help address adaptation measures to prevent adverse health effects in vulnerable communities. Despite this, it is not clear what role EHPs currently play in relation to climate change. The extent to which adaptation programmes have been incorporated into environmental health departments (namely divisions that deal with safety, health and sustainability of local communities) or work programmes for EHPs is largely unknown.

Acknowledging the sizeable portion of the South African population bearing significant ill health burdens [5, 15, 16], as well as those vulnerable to climate change because of their socioeconomic status [6], it is essential to mainstream adaptation measures in EHP work programmes. These measures need to be innovative [17] to ensure that adaptive climate change interventions, such as planting of trees to provide shade during heat waves or installing air conditioning in public buildings, effectively reduce or eliminate climate risks and improve quality of life. EHPs working in the private, public or voluntary sectors are increasingly expected to work towards helping communities in preventing adverse health outcomes and should therefore be knowledgeable of climate change impacts and preventative actions to mitigate adverse effects well in advance [18]. Their efforts may entail reducing air pollution and carbon emissions, preparing for extreme weather events, and identifying environmental health exposure risks that cause such infectious diseases [17]. This study aimed to assess the climate change-related perceptions, knowledge and preparedness of a sample of South African EHPs to identify gaps and opportunities to better inform training and education awareness campaigns among health professionals, as well as consider the role of EHPs in implementing climate change-related adaptation actions in communities.

## Methods

### Study population and study site

The study population comprised EHPs who attended the 2017 South African Institute of Environmental Health (SAIEH) conference for EHPs in Cape Town. The conference was attended by 201 delegates and brings together as many EHPs as possible. Using the conference site, we implemented convenience sampling and encouraged all EHP participants, specifically, field EHPs and Managerial Staff, to participate in the study. Lecturers and South African Local Government Association (SALGA) stakeholders were excluded from the study.

### Data collection

Two study researchers attended the conference and were based at a stall set up in the main foyer of the conference venue. Upon their arrival, all EHPs were invited to participate in the study. Several appeals, for example, recruitment by researchers and announcements at plenary events, were made for EHPs’ participation in the study. Following informed consent, the EHPs completed the questionnaires without assistance from the researchers. Participating EHPs were given a small token of appreciation and their name was also entered into a lucky draw for a small prize at the end of the data collection phase at the conference.

### Questionnaire

The questionnaire was adapted from a similar instrument used in a previous pilot study (Personal communication: N Naicker; unpublished data) conducted among 38 EHPs working in the Johannesburg Metropolitan Municipality Area. Post-piloting questions were refined and grouped into sections. The questionnaire comprised an introductory section on socio-demographics (name of province, district and local authority, age and gender) and 22 climate change-related questions presented in three sections: (1) knowledge, (2) perceptions and (3) preparedness (Table 1). All of the questionnaires were in English.

**Table 1:** Questionnaire items

Socio-demographics	
1	Name of province, district and local authority
2	Age
3	Gender
Knowledge	
1	Highest level of education
2	Current employment
3	Training on climate change and health
4	Understanding of the meaning of climate change
Perception	
1	Whether or not climate change is a threat to public health
2	Ways in which climate change will impact on communities at the local level
3	Perception of climate change impacts within own local community
4	The three largest health burdens faced by communities as a result of climate change
5	The societal group in EHPs jurisdiction most vulnerable to health effects of climate change
6	The degree to which EHPs should be involved in helping communities adapt to climate change
7	The degree to which EHPs should be involved in helping communities adapt to climate change
8	The level at which adaptation to climate change should be addressed (i.e. global, national, provincial or local)
9	A set of 15 possible climate change health-related mitigation and adaptation interventions with a Likert scale response set from 1 for highest priority to 5 for lowest priority
Preparedness	
1	Presence of a climate change and health working group in the department in which the EHP worked.
2	Presence of a climate change and health policy or strategy in place,
3	Budget for climate change and health activities,
4	An official responsible for climate change and health.
5	Have participated in climate change and health adaptation projects as an EHP,

### Data management and statistical analysis

Data were coded from the questionnaires, captured by one data enterer using a double data entry schema and analysed using the Stata Statistical Package 14. Descriptive statistics to describe the perceptions, knowledge and preparedness of EHPs towards climate change and health adaptation were reported as frequencies.

## Results

### Sample description

Of the 201 delegates who attended the conference, 97% were EHPs. The in-depth questionnaire was completed by 70 EHPs, however, four EHPs did not meet the criteria as they were either stakeholders of SALGA or lecturers. This represented a response rate of 68.0% (total number of EHP delegates was given as 97). Because we used a self-reported approach non-response to some of the questions resulted in missing data.

Two-thirds of the respondents were male (66.7%) and most (81.8%) were younger than 54 years of age (Table 2). EHPs in management positions comprised two-thirds of the respondents. Average age of EHPs was 45.2 years (range = 25–60) and two-thirds of the respondents were male (66.7%). Respondents with less than 5 years of EHP work experience and those who had between 5 and 10 years’ experience were equally 40.9%. Most respondents were resident in the city of Cape Town or Western Cape Province. EHPs education level ranged from national diploma to a doctorate with most (45.5%) EHPs having completed a bachelor’s degree.

**Table 2:** Demographics of EHPs (*N* = 66) who participated in the questionnaire survey

Question and responses	Number of participants n (%)
Gender	
Male	44 (66.7)
Female	22 (33.3)
*Missing*	*0 (0.0)*
Age	
Mean (range)	45.2 (25–60)
^a^Current position	
Management	42 (63.6)
Field EHP	24 (36.4)
*Missing*	*0 (0.0)*
Total years practising as an EHP	
< 5 years	27 (40.9)
5–10 years	27 (40.9)
> 10 years	12 (18.2)
*Missing*	*0 (0.0)*
Province of work	
Gauteng	9 (13.0)
Free State	4 (6.0)
KwaZulu-Natal	7 (10.0)
Western Cape	17 (25.0)
Eastern Cape	8 (12.0)
Northern Cape	3 (4.0)
Limpopo	6 (9.0)
North-West	3 (4.0)
Mpumalanga	9 (13.0)
*Missing*	*3 (4.0)*
Highest level of education	
Diploma	11 (16.6)
Bachelor’s degree	30 (45.5)
Honours	9 (13.7)
Masters	14 (21.2)
Doctorate	1 (1.5)
*Missing*	*1 (1.5)*

^a^Management (managerial position in department of EH) and for Field EHP, Field EHP (practitioner tasked with community engagement and spending time in communities)

### EHPs knowledge, understanding and perceptions of climate change and health

Experience of formal and informal training about climate change and health was reported by 48 EHPs with 53.0% stating conferences/seminars as the source of that training (Table 3). Majority (*n* = 57) agreed that they had noticed climate change effects occurring in their local communities. These effects included drought (e.g. impacts such as crop failure and food production reduction), direct impacts on human health (e.g. malaria and cholera), flooding, increase in extreme weather events (including fires and thunderstorms), increase in temperature (and heatwaves), sea level rise, air pollution impacts, socio-economic impacts (e.g. unemployment and damage to property) and damage to infrastructure. Some EHPs gave specific examples such as ‘the grass is brownish now even during summer’, and another EHP said ‘there are suddenly floods in some areas of Johannesburg’. Drought and water restrictions were also mentioned by the respondents.

**Table 3:** Participants’ self-reported knowledge and understanding of climate change (*N* = 66)

Question and responses	Number of participants n (%)
Ever received training on climate change and health	
No	17 (25.7)
Yes	48 (72.8)
*Missing*	*1 (1.5)*
Training on climate change and health from ^a^	
Formal training only	9 (19.0%)
Informal training only	13 (27.0%)
Both formal and informal training	26 (54.0%)
*Missing*	*0 (0.0)*
‘Climate change is a serious threat to public health’, do you:	
Strongly agree	54 (82.0)
Agree	10 (15.0)
Disagree	0 (0.0)
Strongly disagree	2 (3.0)
*Missing*	*0 (0.0)*
Have you noticed any climate change effects that occurred in your local communities?	
No	6 (9.0)
Yes	56 (85.0)
Don’t know	2 (3.0)
*Missing*	*2 (3.0)*
To what degree should EHPs be involved in helping communities adapt to climate change?	
Leading role	25 (37.8)
Supportive role	41 (62.2)
Minimal role	0 (0.0)
No role	0 (0.0)
*Missing*	*0 (0.0)*
At what level do you think adaptation to climate change should be addressed?	
Global level	28 (42.0)
National level	5 (8.0)
Provincial level	1 (2.0)
Local level	23 (35.0)
More than one level	8 (12.0)
*Missing*	*1 (1.0)*
What societal group in your jurisdiction do you think is most vulnerable to health effects of climate change? (ranked by most reported) ^a^	
Lower income / informal / poor communities	38 (25.3)
Children	34 (22.6)
Elderly people	34 (22.6)
Immuno-compromised people	13 (8.6)
Women	8 (5.3)
People in rural areas	7 (4.6)
Disabled people	5 (3.3)
Black African people	4 (2.6)
Farmers	3 (2.0)
Homeless people	2 (1.3)
*Missing*	*2 (1.3)*

^a^Multiple responses are allowed

### Climate change preparedness in environmental health local and provincial departments

Thirty-nine percent (39.0%) of participants indicated that they had a climate change committee or working group in their departments and 47.0% reported having an official staff member responsible for climate change and health (Table 4). Forty-one percent of EHPs indicated presence of a climate change and health policy or strategy in their departments. About half reported having climate change and health budgets allocated by their departments, however, one in three EHPs confirmed that they had participated in a climate change and health adaptation project in their current position.

**Table 4:** Activities undertaken by EHPs in their workplace in relation to climate change (*N* = 66)

Question and responses	Number of participants n (%)
Does your EH department have a climate and health committee/working group?	
No	29 (44.0)
Yes	26 (39.0)
Don’t know	9 (14.0)
*Missing*	*2 (3.0)*
Does your EH department have a climate and health policy or strategy?	
No	27 (41.0)
Yes	27 (41.0)
Don’t know	10 (15.0)
*Missing*	*2 (3.0)*
Does your EH department have a climate and health budget?	
No	34 (25.8)
Yes	17 (51.5)
Don’t know	14 (21.2)
*Missing*	*1 (1.5)*
Does your EH department have an official responsible for climate and health?	
No	31 (40.9)
Yes	27 (46.9)
Don’t know	8 (12.2)
*Missing*	*0 (0.0)*
Have you ever participated in a climate change and health adaptation project in your current position?	
No	37 (56.0)
Yes	22 (33.3)
Don’t know	3 (4.5)
*Missing*	*4 (6.2)*

### EHPs’ perceptions of mitigation and adaptation interventions

EHPs rated their perceptions of the importance of selected climate and health adaptation interventions from highest to lowest (Table 5). The adaptation measure which the majority ranked highest was ‘scaling up the planting of trees to provide shade during heat waves’ (61.1%). Other interventions with high priority ratings included ‘promoting vegetable gardening’ (59.0%), ‘instituting community-based early warning systems for adverse weather events’ (56.0%) and ‘raising standards for storm water drainage’ (54.5%). ‘Provision of public drinking water’ (37.8%), ‘water fountains in schools’ (42.2%) and ‘shade in school playgrounds’ (45.4%) were also ranked relatively high compared to other options. ‘Air conditioning at schools and churches during heat waves’ (25.7%) and ‘swimming pools’ (13.6%), as well as ‘cycling facilities (24.2%) were given the lowest ratings.

**Table 5:** Proportion of participants’ responses to the rating of climate and health mitigation and adaptation interventions (*N* = 66)

	^b^Proportion of participants (*N* = 66) n (%)				
	Highest priority (1)	(2)	(3)	(4)	Lowest priority (5)
Providing public drinking water fountains	25 (37.8)	14 (21.4)	17 (25.8)	4 (6.0)	6 (9.0)
Providing water fountains in school playgrounds	28 (42.2)	11 (16.8)	17 (25.9)	4 (6.1)	6 (9.0)
Providing shade in school playgrounds	30 (45.4)	17 (25.7)	12 (18.4)	1 (1.5)	6 (9.0)
Building bicycling lanes in local communities	20 (30.3)	8 (12.1)	20 (30.3)	6 (9.0)	12 (18.3)
Providing bicycles to poor communities	16 (24.2)	12 (18.2)	14 (21.2)	12 (18.2)	12 (18.2)
Scaling up the planting of trees to provide shade during heat waves	41 (61.1)	8 (12.3)	7 (10.8)	4 (6.4)	6 (9.4)
Providing swimming pools at schools and in communities	9 (13.6)	12 (18.2)	20 (30.0)	16 (24.4)	9 (13.8)
Promoting vegetable gardening	39 (59.0)	14 (21.2)	5 (7.5)	2 (3.3)	6 (9.0)
Promoting insulation of houses^a^	26 (39.3)	23 (35.1)	11 (16.9)	3 (5.4)	2 (3.3)
Raising standards for storm water drainage	36 (54.5)	14 (21.3)	11 (16.6)	0 (0.0)	5 (7.6)
Lobbying for climate conscious housing settlements (e.g. walkability, dwelling orientation, insulation and tree planting)	32 (48.4)	18 (27.2)	9 (13.6)	0 (0.0)	7 (10.8)
Strengthening climate and health research programmes	34 (51.5)	20 (30.3)	5 (7.5)	2 (3.0)	5 (7.7)
Instituting community-based early warning systems for adverse weather events.	37 (56.0)	17 (25.7)	6 (9.0)	2 (3.0)	4 (6.3)
Public education programmes (e.g. importance of buying locally produced goods) ^a^	32 (48.4)	12 (18.4)	15 (23.4)	1 (1.5)	5 (8.3)
Providing a public building (e.g. hall, recreation centre or school) with air conditioning for vulnerable groups during heat waves.	17 (25.7)	16 (24.2)	20 (30.3)	7 (10.6)	6 (9.2)

Note. ^a^1 value missing. ^b^ According to a Likert scale from highest priority (scored 1) to lowest priority (scored 5)

## Discussion

EHPs are located at the interface between government and communities, positioning them potentially well in relation to climate change adaptation and mitigation implementation. With first-hand community engagement, EHPs can contribute to the shaping of national and local strategies, policies and regulations aimed at supporting vulnerable communities to adapt to climate change. Our study results highlight that although EHPs believe they should be playing a role in climate change adaptation, two-thirds believed that this role should be supportive, rather than leading. Many EHPs (42.0%) reported that climate change adaptation action was best undertaken at a global level suggesting that EHPs may not fully identify their role in climate change adaptation among communities.

In South Africa, the National Climate Change Response White Paper and the Climate Change and Health Adaptation Plan provide a useful framework for EHP action at local level [13, 14]. However, the details of an integrated and holistic local level climate and health adaptation strategy need to be developed and implemented nation-wide. Presently comprehensive information on community level policy, the role of inter-sectoral action, regulations and standards, budgetary matters, research, training and education in relation to climate change and health is lacking [19]. In the current study, while two in three environmental health departments had institutional arrangements in place to support EHP actions on adaptation to climate change there were several potential barriers such as budgetary and resource constraints. Some municipalities did have climate change and health strategies or policies in place, for example, Western Cape (City of Cape Town) and KwaZulu-Natal (eThekwini Municipality), however, capacity and financial constraints were identified as challenges.

In the current study, most EHPs had received tertiary training of at least a diploma or bachelor’s degree, illustrating their capability to address climate-related health impacts with knowledge and understanding. One in two EHPs reported having had both formal and informal training on climate change, however, biases and inaccuracies may exist in the knowledge and skills of the EHPs who received informal training which may include listening to friends and reading non-peer-reviewed internet sources. Formal EHP training on climate change and environmental health is essential and should be consistently included in the EHP formal curriculum and training at higher degree institutions. It is unclear from the formal environmental health curriculum taught across multiple institutions and in varying formats exactly what is covered on climate change-related health impacts and adaptation measures. It is likely to be a high-level overview; therefore, further research is needed to gauge what subject matter is taught and / or required for teaching EHPs on this important public health issue. In terms of the capacity of EHPs to help communities adapt to climate change, the World Health Organization suggests there should be a ratio of 1 EHP/10000 population [20]. South Africa has adopted a national ratio of 1 EHP/15000 population [20]. In 2016 the public sector EHP/population ratio was 1:78000 (calculated using 2016 on Statistics South Africa population data). This presents a multi-pronged barrier towards designing and implementing adaptation strategies as human resources are essential in managing climate change at community-level.

EHPs highlighted several adaptation measures, such as providing access to air conditioning in public buildings as effective factors for controlling heat-related stresses [21]. They also recommended heat emergency plans detailing the human and material resources needed to reduce or manage risks associated with weather-related disasters [21]. For EHPs to fully realize their potential to help communities adapt to climate change, there needs to be an inclusive discussion on the nature of the role they might play, and the essential resources needed to support their efforts. Inspiration might be taken from some encouraging initiatives currently underway. For example, the City of Rustenburg, in partnership with the South African Medical Research Council, is currently in the process of developing a Heat and Health Plan for its citizens. The effort was prompted by the designation of Rustenburg as a vulnerable location [22] when, in 2016, during a single heatwave tens of people died in the North-West province. The climate and health adaptation measures suggested during a consultative workshop in Rustenburg echo those mentioned in the current study in which EHPs rated most highly tree planting, vegetable gardening, strengthening of policies and standards for storm water drainage, strengthening climate and health research programmes, and developing community early warning systems. On the other hand, the provision of cool spaces (air conditioning at schools and churches for example) and swimming pools, as well as cycling facilities, were given the lowest ratings. These adaptation mechanisms may have received the lowest ratings because they are uncommon occurrences in informal rural and low socio-economic settlements and may not be perceived as viable and affordable options.

Implications of our findings point to a specific set of capabilities of EHPs which set them apart as agents of change compared to other public health practitioners. Our findings suggest that involvement of EHPs may be means to foster climate change adaption measures in communities. However, without defined and clear involvement, budget, education and practice to recognise the role of EHPs, the impact of climate change will continue to fall heavily on families living in the local communities. It is in the interests of both the environmental health profession and vulnerable communities throughout South Africa, for EHPs, without delay, to make a concerted effort to define and elaborate on the roles they intend to play in climate change and health adaptation in the country. However, this requires political will, operational policy, clear leadership and workforce development strategies from the national level. This study contributes to a platform of knowledge and understanding that may inform EHPs’ thinking, planning and improve climate change governance.

Despite our attempts to bolster the sample size, our study sample was limited in that we used a conference as a venue to collect questionnaire responses from conference attendees. EHPs in South Africa are geographically dispersed across the country making them difficult to access, particularly when they are in the field and without Internet access. The conference seemed a convenient way to access a large group of EHPs. However, we did not have a representative sample of EHPs across all provinces of South Africa, since costs for travel to the conference venue from distant provinces may have limited participants from those provinces attending. We did try to incentivise participation by having a lucky draw for all respondents, however, respondents were likely those individuals who were interested in the topic which may skew our findings. Future studies should consider alternative sampling mechanisms, besides using a convenience sample at a conference, to gather responses from a fully representative sample. This might entail an online, mobile phone application or telephonic survey. Since the conference was held in Cape Town, majority of conference participants were based in Cape Town hence EHPs working in the Western Cape were oversampled. However, out of the eight metropolitan municipalities in South Africa, six metropolitan municipalities had representatives at the conference therefore ¾ of the major urban areas in the country were included. Of the 44 district municipalities nationwide, more than half (*n* = 24) of them had representatives who participated in the study. Despite this, there was insufficient variation among the study sample to permit sub-analysis of the study findings. For example, most of the EHPs had tertiary education of similar levels, limiting analysis by education level and inferring that results likely pertain mostly to EHPs with similar education as the EHPs who responded to our questionnaire.

The questionnaire was a combination of closed and open questions which allowed for both quantitative and qualitative analyses of findings. It did also make the questionnaire relatively long and may have discouraged participation. It may have been more insightful to have used a qualitative research method and get detailed information from key stakeholders rather than aim for a representative sample. There are limited studies on the perceptions, knowledge and preparedness of EHPs with regards to climate change and health adaptation. Therefore, despite the small sample size, this study adds to the body of evidence on the perceptions of EHPs and the important role they should have to curb adverse climate change-related human health impacts.

## Conclusions

EHPs have a key role to play in resolving environmental challenges and preventing diseases of environmental origin to ensure healthy and safe environments for communities. Therefore, implementation of climate change adaptation plans should be incorporated within the EHPs Scope of Practise. EHPs acknowledge that climate change poses a serious threat to public health and they largely agree that they should play a supportive or leading role in helping communities adapt to climate change. Their efforts require the support of government management at all levels of governance. For environmental health departments to fully implement climate change and health adaptation measures and strengthen the national plans for climate change, adequate human and financial resources should be provided and trickle down to where EHPs need them to be effective ‘agents of change’ among communities. As climate change occurs, EHPs will potentially have an important role to play in ensuring that the environments (especially built environments) in which people live, learn and play, are fit to promote and support health and safety. Owing to the spatial variability in climate change, the tasks of coping with its effects fall on EHPs as local government public practitioners.

## Abbreviations

COP: Conference of the Parties
EHPs: Environmental Health Practitioners
HPCSA: Health Professions Council of South Africa
IPCC: Intergovernmental Panel on Climate Change
NDOH: National Department of Health
SAIEH: South African Institute of Environmental Health
SALGA: South African Local Government Association
